# Transcriptomic analysis identifies novel genes and pathways for salt stress responses in *Suaeda salsa* leaves

**DOI:** 10.1038/s41598-020-61204-x

**Published:** 2020-03-06

**Authors:** Xuejie Zhang, Yan Yao, Xiaotong Li, Luoyan Zhang, Shoujin Fan

**Affiliations:** Key Lab of Plant Stress Research, College of Life Science, Shandong Normal University, Jinan, 250014 Shandong China

**Keywords:** Plant signalling, Salt

## Abstract

Salinity is a critical abiotic stress, which significantly impacts the agricultural yield worldwide. Identification of the molecular mechanisms underlying the salt tolerance in euhalophyte *Suaeda salsa* is conducive to the development of salt-resistant crops. In the present study, high-throughput RNA sequencing was performed after *S. salsa* leaves were exposed to 300 mM NaCl for 7 days, and 7,753 unigenes were identified as differently expressed genes (DEGs) in *S. salsa*, including 3,638 increased and 4,115 decreased unigenes. Moreover, hundreds of pathways were predicted to participate in salt stress response in *S. salsa* by Gene Ontology (GO), MapMan and Kyoto Encyclopedia of Genes and Genomes (KEGG) enrichment analyses, including ion transport and sequestration as well as photoprotection of photosystem (PS) II. The GO enrichment analysis indicated that genes related to ion transport, reactive oxygen species (ROS) scavenging and transcriptional factors were highly expressed upon NaCl treatment. The excessive Na^+^ and Cl^−^ ions were supposed to be absorbed into the vacuole for ion sequestration and balance adjustment by potassium transporters (such as KEA3) with high expressions. Moreover, we predicted that mutiple candidate genes associated with photosynthesis (such as PSB33 and ABA4), ROS (such as TAU9 and PHI8) and transcriptional regulation (HB-7 and MYB78) pathways could mitigate salt stress-caused damage in *S. salsa*.

## Introduction

Salt stress, one of the most important abiotic factors, greatly affects global agricultural productivity. It is estimated that about 6.0% of the land worldwide, more than 800 million ha, is either salt affected or has been subjected to soil salinization^1,2^. The food production is increased at a rate of 1.8% per year, the world population is expected to exceed 9.5 billion people by 2050 (http://www.fao.org/wsfs/world-summit/en), and 30% of the cultivable soils will become unusable due to salt stress, all of which pose serious challenges to salt alleviating technologies for agricultural research^3^.

Plants can be divided into halophytes and glycophytes according to the ability of salt tolerance. External salt concentration of 86 mM NaCl has been taken as a general limit, above which the plant yield of glycophytes is severely reduced^4,5^. Nevertheless, halophytes can grow in a surrounding containing even 200 mM NaCl or more, and they are further categorized into euhalophytes, recretohalophytes and pseudo-halophytes^6–9^. Salt-diluting halophytes, such as sea beet and other succulent halophytes, are able to balance low external water potential and generate turgor by accumulating high contents of internal Na^+^ and Cl^−^ ions in their vegetative tissues^10–12^. Recretohalophytes are typical halophytes, which have developed a series of adaptive secretory structures, including salt glands and salt bladders, for salt excretion^2,4,5,13–15^. These characteristic visible structures of recretohalophytes play a crucial role in excreting excess salt out of the plants to avoid ionic damage^13,14,16–20^.

Euhalophytes with salt-diluting ability accumulate high concentrations of salt in their cells and tissues and overcome salt toxicity by developing succulence^6,8,13,21–24^. These euhalophytes include *Excoecaria agallocha*, *Limnitzera racemosa*, *Sonneratia acida*, *Salvadora persica*, *S. nudiflra*, *S. nudiflra*, *S. maritima*, *S. salsa* and *Pentatropis sianshoides*^7,14,25,26^. The excessive Na^+^ and Cl^−^ ions in the vacuole can be compartmentalized by euhalophytes, which helps to lower the water potential of the cells and tissues and subsequently enhance water absorbance from the saline soil to attenuate the ion concentration in the cytoplasm that is detrimental for enzyme activity and biological substances^27–29^. Besides, accumulation of compatible solutes has been regarded as one of basic strategies for the protection and survival of euhalophytes under salt stress, including soluble carbohydrates, glycocoll betaine, polyols and proteins. These compatible solutes protect plants against stress by adjusting cellular osmotic pressure, detoxifiation of reactive oxygen species (ROS), maintaining membrane integrity, and stabilizing enzyme and protein complex^30–35^.

In our current work, we examined the responses of *S. salsa* to salt stress using Illumina sequencing. This species is a leaf-succulent C3 halophytic herb and becomes one of the most important halophytes in China because of its ability of high salt tolerance^21,36–38^. Besides, the species is of great economical value, because its seeds not only contain 40% oil, but are rich in unsaturated fatty acids, which can be easily converted to chemical compounds for industrial uses^21,36,37,39–41^. *S. salsa* has adapted to the high salinity in northern China, and it is native to saline soils. The optimal NaCl concentration for its growth is 350 mM, making it well-suited for studies on the response to salt stress as well as for the physiological and molecular characterization of salt tolerance in plants^37,38^. Genes important for the vacuolar compartmentalization of Na^+^ have been cloned, and their functions have been tested in *S. salsa*, such as NHX Na^+^/H^+^ antiporters *SsNHX*, vacuolar H^+^-ATPase (V-H^+^-ATPase) genes, *SsVHA-H* and *SsVHA-B*^23,29,33,42–45^. Many previous studies have shown the responses of *S. salsa* under salt stress. However, the underlying mechanisms of salt deposition via ion compartmentalization, osmotic adjustment and transcriptional regulation remain largely unknown.

As an effective strategy, transcriptome sequencing can be used to detect candidate participants of stress response on a genome-wide scale. A great deal of attention has been paid to salt stress responses in halophytes using sequencing technologies^2,46–56^. Full-genome transcriptome analysis through deep sequencing seems to be the most promising strategy to identify candidate genetic determinants of salt tolerance in *S. salsa*. Therefore, we attempted to explore the essential salt responding genes and their modulators in *S. salsa* in the present study.

## Results

### Transcriptome profiling of *S. salsa*

A total of 474,575,48, 431,877,04, 445,313,26, 434,315,68, 536,186,10 and 405,137,42 paired-end reads were obtained from three control and three salt-treated leaf samples of *S. salsa* through sequencing by the Illumina HiSeq X platform (Table 1). In addition, *de novo* transcriptome assembly generated 86,255 unigenes, the average length and N50 of which were 1,647 nt and 2,243, respectively. Generally speaking, 79.16% of the reads were mapped to the reference genome (Table 1).

**Table 1:** Summary of mapping transcriptome reads to reference sequence in *Suaeda salsa*.

Sample name	Sample description	Total reads	Total mapped	Ratio of mapped reads
C_0_1	Control replication 1	474,575,48	378,159,82	79.68%
C_0_2	Control replication 2	431,877,04	328,783,64	76.13%
C_0_3	Control replication 3	445,313,26	348,040,16	78.16%
S_300_1	Salt stress replication 1	434,315,68	347,175,14	79.94%
S_300_2	Salt stress replication 2	536,186,10	432,269,66	80.62%
S_300_3	Salt stress replication 3	405,137,42	325,874,04	80.44%

### Functional annotations of unigenes in *S. salsa*

**Figure 1:**
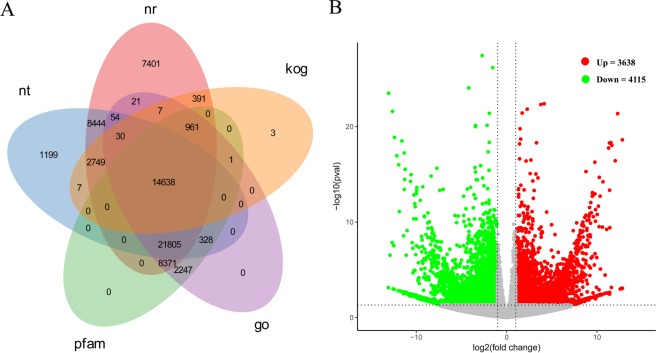
(**A**) Venn diagram of functional annotations of unigenes in nt (NCBI non-redundant protein sequences), nr (NCBI non-redundant protein sequences), kog (Clusters of Orthologous Groups of proteins), go (Gene Ontology) and pfam (Protein family) databases. (**B**) Expression patterns of differentially expressed genes (DEGs) identified between 300 mM NaCl treated and control. Red and green dots represent DEGs, grey dots indicate genes that were not differentially expressed. In total, 7,753 unigenes were identified as DEGs (padj <0.05) between NaCl treated and control samples, including 3,638 upregulated genes and 4,115 downregulated genes.

Similarity test was carried out to annotate unigenes against different databases by BLASTX. All 86,255 (100%) unigenes were annotated in at least one database. A total of 64,872 (75.20%) (Online Resource 1), 49,254 (57.10%) and 48,351 (56.05%) unigenes showed demonstrated similarity to sequences in NT, NR and PFAM databases, respectively, with an E-value threshold of 1e^−5^ (Fig. 1A). Moreover, we assigned 48,463 (56.18%) unigenes, with an E-value cutoff of 1e^−6^, in Gene Ontology (*GO*) database using Blast2GO v2.5. Besides, 59,738 unigenes of the *S. salsa* were assigned to *A. thaliana* gene IDs for GO annotation mapping by BLASTX with an E-value cutoff of 1e^−5^ and categorized into GO terms for GO analysis.

### Identification of differentially expressed genes (DEGs) in *S. salsa*

The relative expression levels of genes in *S. salsa* under control or salt stress condition were evaluated based on the fragment per kilobase of exon model per million mapped read (FPKM) values, which were calculated based on the uniquely mapped reads. The FPKM values for different genes in six samples ranged from 0 to 74,138.71, with a mean value of 12.47. Furthermore, 3,638 unigenes were filtered as up-regulated genes, and the expressions of 4,115 unigenes were decreased in NaCl-treated samples by comparative analysis when a cutoff of adjusted *P* value (Padj) <0.05 and |log2fold change [L_2_fc]|> 1 were used (Fig. 1B; Table S1).

### GO, MapMan and ***KEGG*** enrichment results of DEGs in *S. salsa*

To uncover the molecular mechanisms underlying the salt tolerance of *S. salsa* leaves, the DEGs were characterized with GO databases. Consequently, 171 biological process (BP) terms were enriched by 3,638 up-regulated unigenes with the cutoff of *P* value < 0.05, including “calcium ion transmembrane transport” (GO: 0070588), “phosphate ion homeostasis” (GO: 0055062) and “cellular protein catabolic process” (GO: 0044257) (Tables 2; S2). The 4,115 genes with decreased expressions were identified to be enriched in 208 BP terms, such as “translation” (GO: 0006412), “polyamine catabolic process” (GO: 0006598) and “photosynthesis, light harvesting in photosystem I” (GO: 0009768) (Tables 2; S2). The DEGs were dispatched to 1,595 and 2,328 homologs in Arabidopsis, respectively. We assigned these genes to 905 pathways by MapMan. Among these pathways, the dysregulated genes were enriched in 55 pathways, with a cutoff of P value < 0.05.

**Table 2:** Top30 biological processes enriched by the up- and down-regulated genes in *S. Salsa*.

GO ID	GO term	Annotated gene number	Enriched gene number	P value
**Up-regulated**				
GO:0070588	calcium ion transmembrane transport	126	23	2.40E-08
GO:0055062	phosphate ion homeostasis	82	18	6.60E-08
GO:0044257	cellular protein catabolic process	1281	74	8.00E-07
GO:2000185	regulation of phosphate transmembrane transport	9	6	8.20E-07
GO:0043487	regulation of RNA stability	78	14	9.60E-07
GO:0007165	signal transduction	4216	249	2.50E-06
GO:0006552	leucine catabolic process	58	12	1.40E-05
GO:0009646	response to absence of light	128	17	5.60E-05
GO:0010252	auxin homeostasis	95	14	1.50E-04
GO:0006970	response to osmotic stress	1872	113	1.70E-04
GO:0040009	regulation of growth rate	35	8	1.80E-04
GO:0031146	SCF-dependent proteasomal ubiquitin-dependent protein catabolic process	133	17	1.90E-04
GO:0010555	response to mannitol	51	11	2.20E-04
GO:0048278	vesicle docking	129	11	2.80E-04
GO:0009734	auxin-activated signaling pathway	432	37	3.10E-04
**Down-regulated**				
GO:0006412	translation	1928	183	1.10E-19
GO:0006598	polyamine catabolic process	22	10	5.80E-09
GO:0070544	histone H3-K36 demethylation	17	10	6.60E-09
GO:0009812	flavonoid metabolic process	292	45	9.10E-08
GO:0042254	ribosome biogenesis	1069	115	5.10E-07
GO:0009768	photosynthesis, light harvesting in photosystem I	88	19	7.20E-07
GO:0009970	cellular response to sulfate starvation	21	9	1.30E-06
GO:0006270	DNA replication initiation	91	19	2.80E-06
GO:0006564	L-serine biosynthetic process	9	6	3.00E-06
GO:0055114	oxidation-reduction process	4069	299	4.40E-06
GO:0009813	flavonoid biosynthetic process	174	29	4.90E-06
GO:0042752	regulation of circadian rhythm	192	28	5.30E-06
GO:0002181	cytoplasmic translation	61	14	1.10E-05
GO:0006268	DNA unwinding involved in DNA replication	54	13	1.10E-05

**Figure 2:**
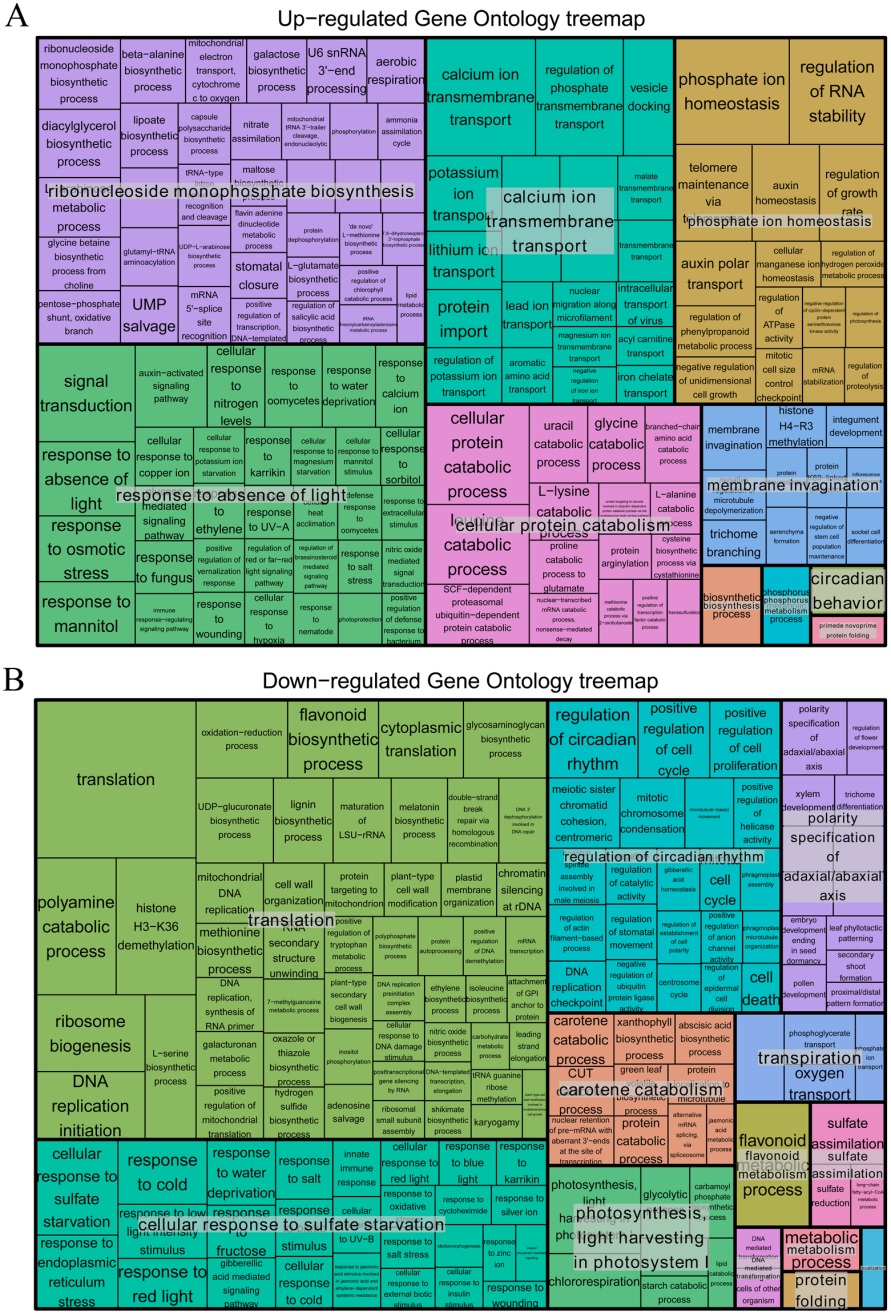
(**A**) REVIGO analysis results for genes up-regulated in *S. salsa* under salt stress. (**B**) REVIGO analysis results for genes down-regulated in *S. salsa* under salt stress. Each rectangle is a single cluster representative. The representatives are joined into “superclusters” of loosely related terms, visualized with different colors. Size of the rectangles was adjusted to reflect the *P value* of the GO term calculated by TopGO. In this study, 171 up-regulated processes were integrated into 10 groups, 13 subsets were summarized for 208 biological processes enriched by down-regulated genes.

Due to the large numbers and complex branch structures of GO categories, REVIGO was used to find representative subgroups of the terms using a simple clustering algorithm that relies on semantic similarity measures. The 171 BP terms enriched by genes with increased expressions were integrated into 10 groups (Fig. 2A), 37 terms were dispatched to “ribonucleoside monophosphate biosynthesis” subset, and 32 terms were classified to “response to absence of light” group. Moreover, 208 BP terms enriched by genes with decreased expressions were dispatched into 10 subsets (Fig. 2B), such as “translation” (including 52 terms) and “cellular response to sulfate starvation” (including 27 terms).

**Figure 3:**
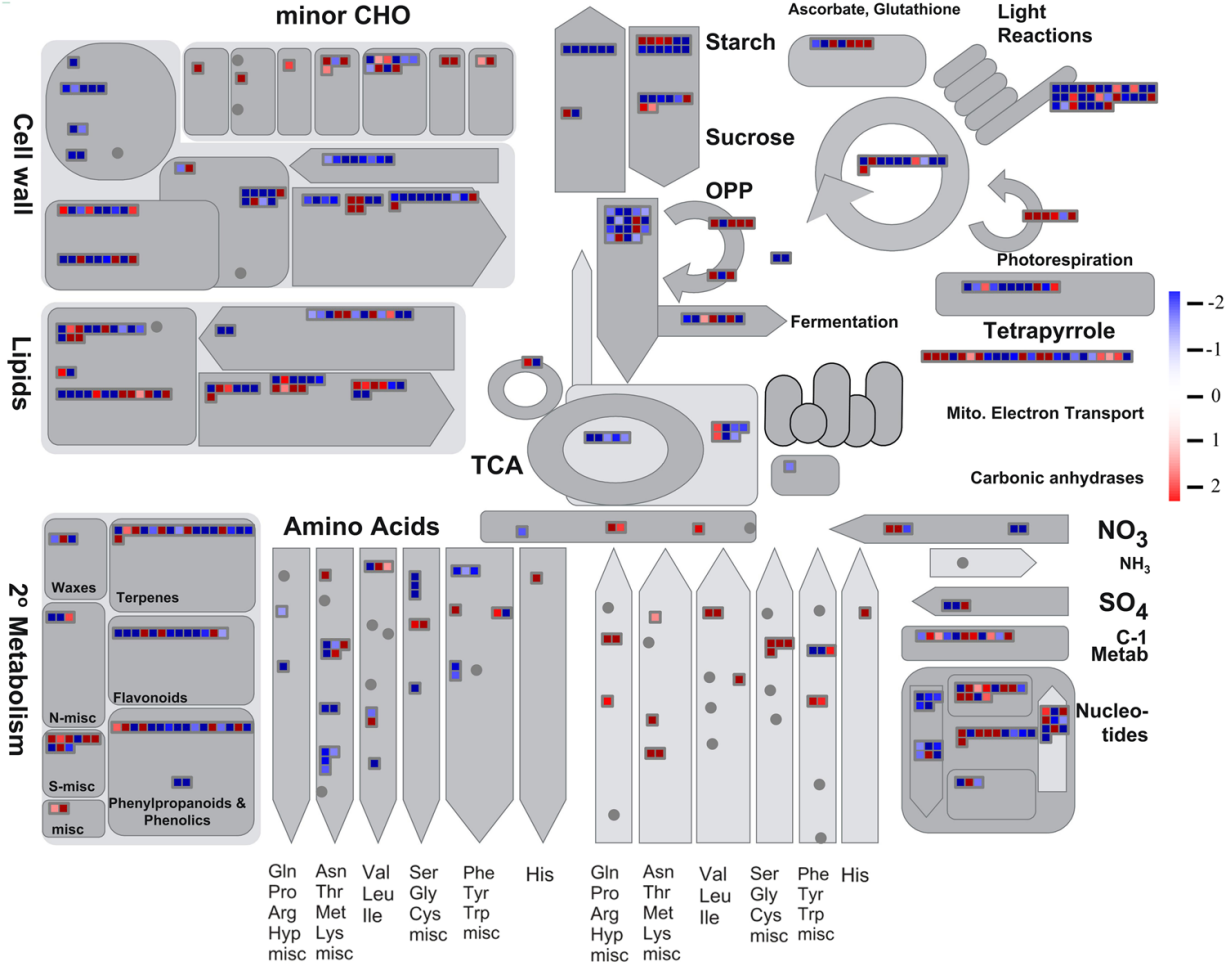
Global view of differently expressed genes (DEGs) involved in diverse metabolic pathways. DEGs genes were selected for the metabolic pathway analysis using the MapMan software (3.5.1 R2). The colored boxes indicate the Log_2_ of expression ratio of DEGs genes. The dys-regulated unigenes were assigned to 1,595 and 2,328 homologs in Arabidopsis, respectively. These genes were mapped to 905 pathways by MapMan, of which, 55 pathways were filtered enriched by the dys-regulated genes with the cutoff p-value <0.05.

Figure 3 illustrates the metabolism result of MapMan analysis. The KEGG pathway “plant hormone signal transduction” (ko04075) was enriched by 23 up-regulated unigenes, of which the cell enlargement-related sub-pathway “tryptophan metabolism” was enriched by auxin-responsive protein (AUXIAA, Cluster-12522.63856, L_2_fc = 1.341), auxin response factor (ARF, Cluster-12522.18986, L_2_fc = 5.928) and SAUR family protein (SAUR, Cluster-1,512.0, L_2_fc = 6.226); the stomatal closure regulation sub-pathway “carotenoid biosynthesis” was enriched by four up-regulated unigenes, including abscisic acid receptor PYR/PYL family protein (PYR, Cluster-12522.12070, L_2_fc = 4.312), protein phosphatase (PP2C, Cluster-12522.20854, L_2_fc = 9.005) and ABA responsive element binding factor (ABF, Cluster-12522.47885, L_2_fc = 2.532).

### The differentially expressed transcription factors (TFs)

In the present study, we found that 2,656 unigenes were dispatched to 47 TF families (Table S3), and 293 differentially expressed TFs were found in NaCl-treated leaf samples, including 145 genes with increased expressions and 148 gens with decreased expressions (Table S3). Of the up-regulated TFs, the largest number of up-regulated genes was found in HB (18 unigenes), followed by MYB (13 unigenes) and bZIP families (10 unigenes). Conversely, the largest number of down-regulated genes was found in MYB (20 unigenes), followed by the bHLH and AP2/ERF families, which contained 13 and 10 unigenes with decreased expressions, respectively (Table 3).

**Table 3:** The TF family and their contained dys-regulated gene number.

TF family	Up gene num.	Down gene num.
MYB	13	20
HB	18	9
bHLH	7	13
AP2/ERF	9	10
bZIP	10	6
C2C2	4	12
GARP	9	5
B3	3	9
MADS	8	4
NAC	7	5
C3H	6	5
C2H2	7	3
NF	8	1
FAR1	7	1
SBP	6	2
GRAS	3	4
TCP	4	3
WRKY	4	2
HSF	2	3
GRF	0	4

Note: Up gene num.= Up-regulated gene number; Down gene num.= Down-regulated gene number.

### Real-time quantitative PCR (RT-qPCR) validation

**Figure 4:**
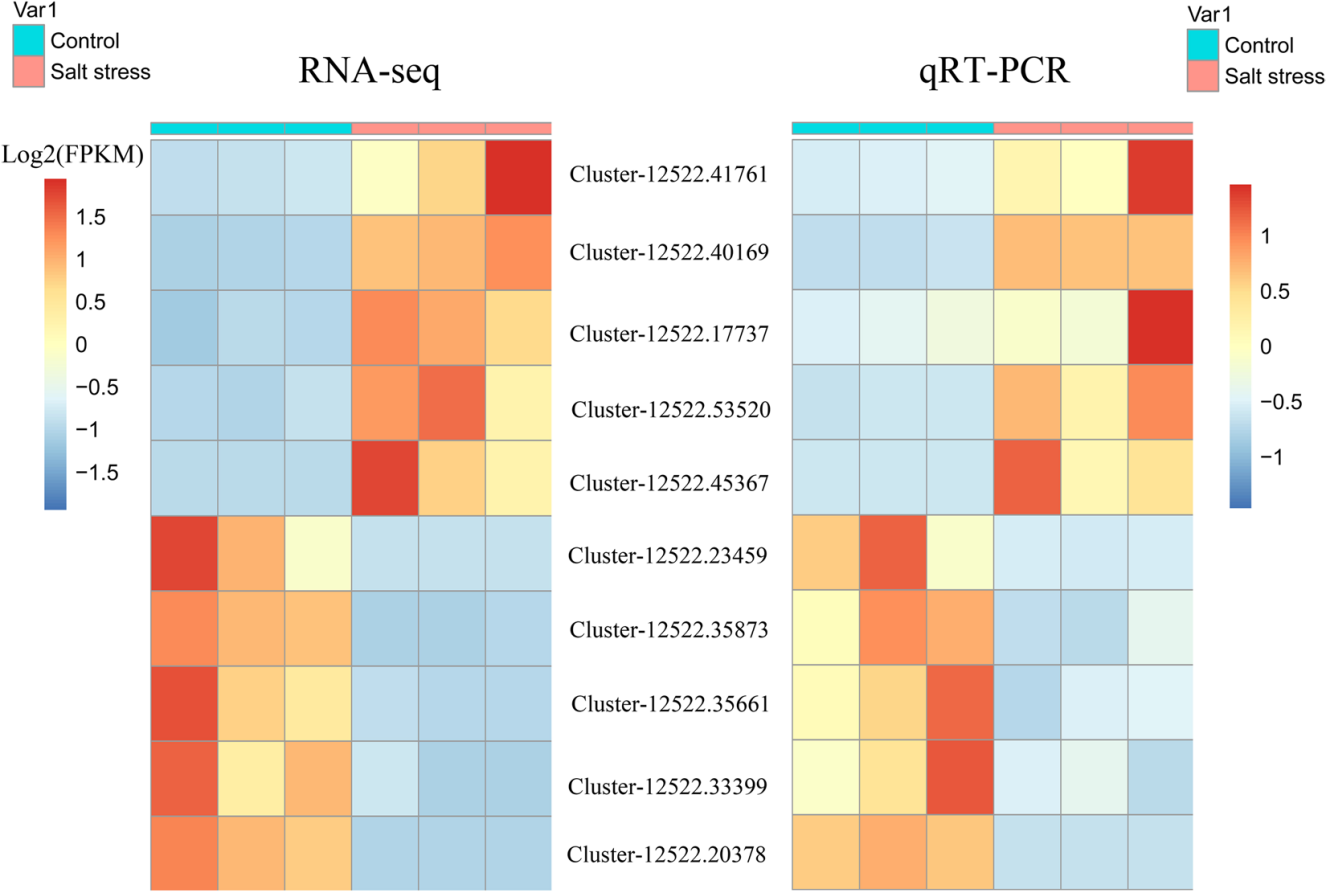
Validation of RNA-Seq results by qRT-PCR using ten *S. salsa* dys-regulated genes. The expression levels of ten selected differentially expressed genes under control and salt stress are shown. A red color indicates that the gene is highly expressed under the corresponding treatment. Log2(FPKM) means Log2() value of FPKM for unigenes.

To verify the RNA-Seq results, an alternative strategy was selected for the dysregulated unigenes. Four up-regulated and four down-regulated unigenes were selected for RT-qPCR validation with the same RNA samples used for RNA-Seq. Primers spanning exon-exon junctions were designed (Table S4). Figure 4 reveals that these two methods demonstrated similar gene expression profiling in most cases. For example, the ortholog of salt stress responding gene *ITN1*, Cluster-12522.40169, was significantly increased by RT-qPCR method (Fig. 4), and it was also an up-regulated unigene in the salt-treated samples by RNA-Seq (L_2_fc = 1.573).

## Discussion

Among the most serious environmental factors, salt stress greatly affects the development and yield of crops worldwide. External salt concentrations severely inhibit photosynthesis in glycophytes^1,5,57,58^, while halophytes maintain or have enhanced photosynthesis and ion balance at these salt concentrations^36,37,59^. *S. salsa*, a euhalophyte plant with salt-diluting ability, can adapt to high salinity, and its photosystem (PS) II shows high resistance to salt stress^41,44,60–63^. Many previous studies have shown the responses of *S. salsa* under salt stress. However, the underlying mechanisms of salt deposition via ion compartmentalization, osmotic adjustment and transcriptional regulation remain largely unclear. Therefore, we attempted to explore the essential salt responding genes and their modulators in *S. salsa*. Our transcriptome analysis provided valuable insights into the molecular mechanisms of salt tolerance and insulation of photosynthesis in *S. salsa* leaves.

Salt stress induces damage primarily through osmotic and ion stresses. Therefore, halophytes can resist to salt stress due to their ability to adjust osmotic state and ion balance^14,28,64^. The excess Na^+^, Cl^−^ and oxidative stress in the intracellular or extracellular environment activate the osmotic adjustment or homeostasis regulating salt stress responses^27,28^. In salt-diluting halophyte, the vacuole is supposed to absorb excessive Na^+^ and Cl^−^ for ion sequestration and balance adjustment. Na^+^ generally enters cells via ion transporters, such as potassium transporter (KT)^27–29^. Guo *et al*.^65^ have shown that the expression levels of Na^+^/H^+^ exchanger, V-H^+^ ATPase, choline monooxygenase, potassium and chloride channels are up-regulated in *S. salsa* leaves upon 500-mM saline treatment, which can reduce the over-accumulation of Na^+^ and Cl^−^. Our transcriptomic data showed that the expressions of KT-encoding genes were increased to enhance protein yield to absorb more ions, such as K^+^ efflux antiporter 3 (Cluster-12522.45367, L_2_fc = 7.991). Furthermore, calcium ion transmembrane transport participating cation exchanger 3 (Cluster-12522.69849, L_2_fc = 6.425) and 5 (Cluster-12522.41534, L_2_fc = 3.393) were found to be highly expressed under salt stress, indicating their functions in transporting ions in *S. salsa*. Besides, excess Cl- was predicted to be transported by chloride channel B (Cluster-12522.3333, L_2_fc = 3.546) and C (Cluster-12522.30526, L_2_fc = 3.828).

In plants, salt stress-triggered ion injury and osmotic stress usually affect a set of basic biosynthetic functions^27,28^. As one of the most sensitive physiological indices, photosynthesis is susceptible to salt stress^7,44,49,50^. Although *S. salsa* shows significant resistance not only to salinity stress but also to photoinhibition even when exposed to 400 mM NaCl or full sunlight^41,60,61^, the photosynthesis rate is decreased by Cl^−^ treatments^66^. In this study, the BP term “photosynthesis, light harvesting in photosystem I” (GO: 0009768) was enriched by 19 down-regulated genes. For example, photosystem I light harvesting complex gene 1 (Cluster-12522.54286, L_2_fc = −3.698), gene 3 (Cluster-12522.42577, L_2_fc = −2.321) and chlorophyll A/B binding protein 1 (Cluster-12522.36469, L_2_fc = −3.821) were down-regulated under salt stress, indicating the impairment of PSI light harvesting systems in *S. salsa*. Furthermore, photosystem b protein 33 (PSB33) is associated with the chloroplast thylakoid membrane and provides stability to PSII in Arabidopsis^39,67^. In the present study, homolog of PSB33 (Cluster-12522.32409, L_2_fc = 9.833) was up-regulated in NaCl-treated leaves in *S. salsa*. Besides, neoxanthin acts as an antioxidant within the PSII supercomplex in thylakoids, involving in the photoprotection of PSII^67^. In this study, the expressions of neoxanthin biosynthesis-required gene abscisic acid-deficient 4 (ABA4, Cluster-12522.63197, L_2_fc = 1.103) was increased when exposed to salt stress. Our transcriptomic data provided solid evidence supporting the tolerance of PSII to salt stress for euhalophyte *S. salsa*^68^.

Saline stress inevitably results in the ROS overproduction in plants. Therefore, the activation of the ROS scavenging system is necessary for the plant to avoid additional oxidative stress^69,70^. An effective enzymatic antioxidant defense system has been evolved by plants for scavenging ROS^71–73^. In this study, the expressions of ascorbate peroxidase (APX), glutathione S-transferase (GST) and glutathione peroxidase (GPX) were increased to varying degrees to avoid oxidative stress induced by salinity, including ascorbate peroxidase 1 (Cluster-12522.1335, L_2_fc = 9.348), glutathione S-transferase TAU9 (Cluster-12522.44190, L_2_fc = 4.923), glutathione S-transferase PHI8 (Cluster-12522.25968, L_2_fc = 8.422) and glutathione peroxidase 4 (Cluster-12522.20682, L_2_fc = 8.949). Our transcriptomic data showed that *S. salsa* leaves could resist the oxidative stress caused by high salinity by recruiting the ROS scavenging system.

TFs play critical roles in salt tolerance in plants via transcriptional regulation of the downstream genes responsible for response to salt challenges. Members of several TF families were verified to control and modulate salt stress adaptive pathways, as bZIP, WRKY, ERF/AP2, HB, MYB and bHLH^74–77^. The HB family TFs with homeodomain-leucine zipper domain are transcriptionally regulated in an ABA-dependent manner and may act in a signal transduction pathway which mediates abiotic stress response. In the present study, 18 and one HB TFs were up-regulated and down-regulated in NaCl-treated samples, respectively, including homeobox 7 (Cluster-12522.23710, L_2_fc = 2.558) and BEL1-like homeodomain 2 (Cluster-12522.57518, L_2_fc = 3.612). The MYB family members have been proved to be key factors in regulatory networks of abiotic stresses^75,77^. In our current study, 13 and 20 MYB family TFs were up-regulated and down-regulated in NaCl-treated samples, respectively, including MYB domain protein 78 (Cluster-10,720.0, L_2_fc = 3.050) and Reveille 8 (Cluster-12522.18843, L_2_fc = 3.777). Numbers of bZIP proteins isolated from diverse species, including Arabidopsis, rice, tomato and halophytes, can enhance the salt tolerance ability^77^. Moreover, 10 bZIP family members were up-regulated in salt-treated samples in this analysis, such as unfertilized embryo sac 12 (Cluster-12522.12729, L_2_fc = 8.992) and iaa-leucine resistant 3 (Cluster-12,694.1, L_2_fc = 2.701). The spatio-temporal gene expression patterns of these TFs and novel genes involved in RNA processing in *S. salsa* could be helpful in understanding the transcriptional regulation in euhalophytes under salt stress.

Plant hormones, especially auxin, ethylene and jasmonic acid signaling transduction pathways, were all up-regulated in response to saline treatment in *S. salsa*, which are important to gene regulations of ion transport and antioxidation^65^. It has been well documented that as an endogenous signaling molecule, ABA enables plants to survive under salt stress^78,79^. In the present study, we investigated the ABA signaling pathway in response to saline stress in the leaves of *S. salsa*, and found that a part of molecular mechanism responding to NaCl could be attributed to the ABA-dependent signaling pathway. The osmotic stress-induced ABA signaling component aldehyde dehydrogenase 7B4 (Cluster-12522.47012, L_2_fc = 7.613) and aldehyde dehydrogenase 3H1 (Cluster-12522.7455, L_2_fc = 1.033)^80^ are dramatically activated in NaCl-treated leaves. Besides, the expressions of MYB/NAC TF family members were increased when exposed to salt stress in this study, including ABA-induced MYB domain protein 78 (Cluster-10,720.0, L_2_fc = 3.050) and NAC domain-containing protein 83 (Cluster-12522.39867, L_2_fc = 1.533). Thus, ABA might exert direct effects on MYB/NAC TFs and modulate salt-resistance in *S. salsa* under salt stress.

## Conclusions

In the present study, a transcriptome analysis was carried out to assess the salt stress responses in *S. salsa* leaves. RNA-Seq revealed that the expressions of 3,638 unigenes were increased when exposed to salt stress, while 4,115 unigenes were down-regulated in *S. salsa*. Moreover, GO, MapMan and KEGG enrichment analyses supported that hundreds of pathways were involved in salt stress response in *S. salsa*, such as ion transport and sequestration as well as photoprotection of PSII. The GO enrichment analysis indicated that genes associated with ion transport, ROS scavenging and TFs were highly expressed under salt stress. The excessive Na^+^ and Cl^−^ were transported into the vacuole for ion sequestration and balance adjustment by highly expressed potassium transporters (such as KEA3). In addition, various candidate genes associated with photosynthesis (such as PSB33 and ABA4), ROS (such as TAU9 and PHI8) and transcriptional regulation (HB-7 and MYB78) pathways were predicted to ameliorate salt stress-induced damage in *S. salsa*.

## Materials and Methods

### Plant material and stress treatments

Seeds of *S. salsa* were collected at the Yellow River Delta, Dongying, China (37 °7′38″N; 118 °41′28″E), in October 2017. The plump seeds were firstly screened, disinfected with 75% alcohol for 2 min, and then washed by water. After vernalization at low temperature (4 °C) for 3 days and germination for 2 days, the seedlings of *S. salsa* were grown in plastic pots (14 cm in diameter and 13 cm in height) filled with sands in a greenhouse under the conditions of 15/9-h light/dark cycle at temperature (28 ± 3/20 ± 3 °C, day/night) with illumination of 600 μmol m^−2^ s^−1^, and half-strength Hoagland nutrient solution was given daily.

No physiological interpretation of the data was included in this study because the design of this study referenced several existing studies demonstrating the changes/regulations of *S. salsa* under salt stress at the morphological and physiological levels. Lu *et al*.^60^ and Sui *et al*.^81^ have demonstrated the physiological meaning of salt treatment/absence for *S. salsa*. Compared with the control (0 mM NaCl treatment) group, the growth of *S. salsa* is significantly increased by salt treatment. Sui *et al*.^81^ have shown that seedlings with 2–3 branches are subjected to 300 mM NaCl treatment, and the growth of *S. salsa* is significantly increased. PSII is rather tolerant to NaCl, and salt stress has no effect on PSII photochemistry in dark-adapted leaves. Photosynthetic electron transport is increased under salt stress. Although 400–500 mM NaCl is regarded as high salinity for *S. salsa*, 300 mM NaCl is the inflection point of the growth, ion accumulation and enzymatic antioxidant defense system for scavenging of ROS^81^. Given these above-mentioned physiological evidence, 300 mM NaCl was selected for salt stress treatment.

Vigorous seedlings were selected and transferred into plastic pots containing well-washed river sand (three plants per pot) when the sixth leaf emerged. NaCl was dissolved in the full-strength Hoagland nutrient solution Plants of control group was irrigated with full-strength Hoagland nutrient solution (0 mM NaCl), while others were exposed to 300 mM NaCl. The NaCl level was gradually increased by 50 mM per day to avoid osmotic shock. After 7 days, the seedlings were selected to determine the indices. Three replicates of each treatment were sampled for transcriptome sequencing.

### Illumina library construction and sequencing

Sequencing libraries were generated using the NEBNext^®^ Ultra^TM^ RNA Library Prep Kit for Illumina^®^ (NEB, USA) based on the manufacturer’s instructions, and index codes were added to attribute sequences to each sample. Briefly, mRNA was purified from total RNA using poly-T oligo-attached magnetic beads. The first-strand cDNA was synthesized using random hexamer primer and M-MuLV Reverse Transcriptase (RNase H^−^). Second-strand cDNA was synthesized using DNA polymerase I and RNase H. The AMPure XP system (Beckman Coulter, Beverly, USA) was employed to purify cDNA fragments of 150~200-bp. The size-selected, adaptor-ligated fragments were purified and enriched by PCR amplification. The resulting products were used for sequencing analysis. The Illumina HiSeq X platform (Illumina, San Diego, CA, USA) was used to perform high-throughput sequencing. The full assembly data were submitted to the NCBI Sequence Read Archive (SRA) database (https://www.ncbi.nlm.nih.gov/sra), SRA accession: PRJNA527358. The processed data files including the assembled sequences and abundance measurements were uploaded to the Gene Expression Omnibus (GEO) database (https://www.ncbi.nlm.nih.gov/geo/), GEO accession: GSE145366.

### De novo assembly of the *S. salsa* transcriptome

RNA sequencing and *de novo* transcriptome assembly were conducted to create reference sequence libraries for *S. salsa*. For each species, an RNA sample of every accession was separately sequenced. cDNA library construction and Illumina paired-end 150 bp sequencing (PE150) were performed at Novogene Co., LTD. (http://www.novogene.com/) according to instructions provided by Illumina Inc. Raw reads of FASTQ format were firstly processed through in-house perl scripts. In this step, clean reads were obtained by removing adapter-containing reads, ploy-N-containing reads and low-quality reads from raw data. Meanwhile, Q20, Q30, GC content and sequence duplication level of the clean data were calculated. All the downstream analyses were carried out based on high-quality clean data. The remaining high-quality reads were used for transcriptome assembly using the Trinity software pipeline with default parameters^82^. Clean data sets of three accessions were pooled for *de novo* assembly and comprehensive sequence library construction in each studied species. *De novo* assembled unigene sequences were used for BLAST searches and annotation against public databases (NR, NT, Swiss-Prot, Pfam, KOG/COG, Swiss-Prot, KEGG Ortholog database and Gene Ontology) with an E-value threshold of 1e^−5^.

### Determination of gene expression in *S. salsa*

Six independent transcript libraries were generated for three accessions of *S. salsa* by a PE150 sequencing analysis. Gene expression levels for all samples were estimated by RSEM^83^. The clean reads were aligned to the *de novo* assembled transcriptome. Gene expression levels in leaf samples of *S. salsa* were calculated by the FPKM method^84^. The expression levels between salt-treated and control samples were compared according to the FPKM values, with a cutoff of Padj <0.05 and L_2_fc >1.

### GO, MapMan and KEGG annotation and enrichment

The unigenes of *S. salsa* were mapped to *A. thaliana* gene IDs by sequence similarity searching against the genome of *A. thaliana* with an E-value cutoff of 1e^−5^. The topGO package of R^85^ was used to perform the GO enrichment analysis for DEGs in NaCl-treated samples. The DEGs in *S. salsa* were annotated onto metabolic pathways using MapMan (version 3.5.1 R2)^86^. The DEGs of *S. salsa* unigene IDs were transferred to the Arabidopsis TAIR locus IDs during the MapMan analysis. The software KOBAS was used to test the statistical enrichment of DEGs in KEGG pathways in *S. salsa*^87^.

### RT-qPCR verification

The expression patterns revealed by the RNA-Seq analysis were validated using the RT-qPCR. The extracted RNA samples were treated with DNaseI and reversely transcribed into cDNA using the PrimeScript RT Reagent Kit with gDNA Eraser (Takara, Dalian, China). Five up-regulated and five down-regulated unigenes in *S. salsa* were selected for the RT-qPCR assay, including Cluster-12522.45367 (L_2_fc = 7.991), Cluster-12522.53520 (L_2_fc = 4.528), Cluster-12522.17737 (L_2_fc = 3.142), Cluster-12522.41761 (L_2_fc = 2.866), Cluster-12522.40169 (L_2_fc = 1.573), Cluster-12522.20378 (L_2_fc = −12.625), Cluster-12522.23459 (L_2_fc = −7.392), Cluster-12522.35873 (L_2_fc = −4.763), Cluster-12522.35661 (L_2_fc = −4.545) and Cluster-12522.33399 (L_2_fc = −3.283). Ortholog of the *A. thaliana* member of actin gene family ACT7 in *S. salsa* (Cluster-12522.36997) was used as the housekeeping gene. Premier 5.0 software was employed to design gene-specific qRT-PCR primers (18–20 bp) (Table S4). qPCR was performed using SYBR Green qPCR Master Mix (DBI, Germany) on an ABI7500 Real-Time PCR System (ABI, USA). Three replicates were performed for each experiment, and the amplification specificity was evaluated by melting curve analysis. Relative expressions of target genes were calculated using the 2^−ΔΔCt^ method. All values were presented as the mean ± standard deviation (SD). Significant differences were determined using GraphPad software (version: 5.0). The t-test was used to analyze the relationship between the expressions of control and NaCl-treated samples.

### Data archiving statement

All genetic data have been submitted to the NCBI Sequence Read Archive (SRA) database (https://submit.ncbi.nlm.nih.gov/subs/sra), PRJNA527358 and the Gene Expression Omnibus (GEO) database (https://www.ncbi.nlm.nih.gov/geo/), GSE145366.

### Consent for publication

We have carefully read and adhered to editorial policies for manuscripts. We declare that the content of this manuscript has not been published or submitted for publication elsewhere.

## Supplementary information

Dataset 1.

Dataset 2.

Dataset 3.

Dataset 4.

Dataset 5.

## Data Availability

We declare that the all data and materials of this manuscript including the supplementary datasets are available to the journal and all readers.
